# Contact-Force-Sensing-Based Radiofrequency Catheter Ablation in Paroxysmal Supraventricular Tachycardias (COBRA-PATH): a randomized controlled trial

**DOI:** 10.1186/s13063-020-4219-1

**Published:** 2020-04-09

**Authors:** Tamas Geczy, Nawin L. Ramdat Misier, Tamas Szili-Torok

**Affiliations:** 1 Thoraxcenter, Department of Clinical Electrophysiology, Erasmus MC, University Medical Center Rotterdam, Postbus 2040, 3000 CA Rotterdam, The Netherlands; 2 Department of Cardiology, Erasmus MC, University Medical Center Rotterdam, dr. Molewaterplein 40, 3015 GD Rotterdam, The Netherlands; 3 Department of Cardiology, Electrophysiology, Rotterdam, The Netherlands

**Keywords:** Ablation, Contact force sensing, Supraventricular tachycardia

## Abstract

**Background:**

Multiple studies have demonstrated the importance of adequate catheter–tissue contact in the creation of effective lesions during radiofrequency catheter ablation. The development of contact force (CF)-sensing catheters has contributed significantly to improve clinical outcomes in atrial fibrillation. However, CF-sensing technology is not used in the ablation of paroxysmal supraventricular tachycardia (PSVT). The possible reason for this is that PSVT ablation with the conventional approach (i.e. nonirrigated, non-CF-sensing catheters) is considered a relatively low-risk procedure with fairly high success rates (short and long term). The aim of this study is to determine whether CF sensing can further improve the outcomes of PSVT ablation.

**Methods/design:**

The COBRA-PATH study is a single-center, two-armed, randomized controlled trial. Patients without structural heart disease being referred for electrophysiology study, because of PSVT and potential treatment with radiofrequency (RF) catheter ablation, will be randomly assigned to either manual ablation with standard nonirrigated ablation catheters or manual ablation with an open-irrigated ablation catheter equipped with CF sensing (used in a virtual nonirrigated modus). The primary study endpoint is the difference in the number of RF applications during the ablation of atrioventricular nodal re-entry tachycardia, and that of Wolff–Parkinson–White syndrome and atrioventricular re-entrant tachycardia. Secondary outcome parameters include acute and long-term procedural success rates, overall duration of RF applications, procedure/fluoroscopy durations and safety parameters.

**Discussion:**

We expect to see a reduced number/duration of RF applications required to achieve effective lesion creation, and consequently a decrease in total procedure/fluoroscopy times. Although a significant improvement in procedural success rates (acute/long term) might not be feasible to demonstrate (given the relatively high success rate of the standard ablation method), the possible decrease in procedure duration and the consequential reduction of radiation exposure has important clinical implications for both operators and patients undergoing the procedure.

**Trial registration:**

ClinicalTrials, NCT04078685. Retrospectively registered on 2 September 2019.

## Background

Contact force (CF) is a relatively new technology that provides real-time feedback on catheter–tissue contact during radiofrequency (RF) ablation. There is compelling evidence that CF between the tip of the catheter and the target tissue within the cardiac chamber is a key factor for safe and effective lesion creation. Insufficient CF leads to ineffective lesion formation whereas excessive CF may result in procedural complications. Before the development of CF-sensing ablation catheters, operators could only rely on surrogate indicators of tissue contact, such as tactile feedback, intracardiac electrograms, baseline impedance, impedance change, electrode temperature and catheter location by fluoroscopy or electroanatomic mapping. These measures were less accurate and did not provide instant feedback on contact [1–3].

Since the introduction of CF-sensing catheter ablation therapy, many studies have demonstrated the importance of this novel technology in the field of atrial fibrillation ablation, as presented in Table 1 [1, 4–19]. These investigations demonstrated that CF-sensing improves the recurrence rate, significantly reducing the total procedure, ablation times and fluoroscopy times in comparison with conventional therapy. Moreover, based on the findings of these clinical trials, important CF parameters have been determined and introduced into clinical practice guidelines for atrial fibrillation ablation, such as minimum CF, target CF, minimum force–time integral (FTI), continuity index (CI) and lesion-size index (LSI) [7, 17, 19].

**Table 1:** Clinical studies on catheter ablations with contact force (CF)-sensing catheters in patients with atrial fibrillation

Study	Year	Type of study	Number of patients		Follow-up (months)	Ablation	CF catheter
			CF	Control			
Martinek et al. [4]	2012	Prospective nonrandomized study	25	25	n/a	Circumferential PVI	ThermoCool SmartTouch
Kuck et al. [5] (TOCCATA)	2012	Prospective nonrandomized study	72	n/a	12	Circumferential PVI Right-sided SVT ablation	TactiCath
Reddy et al. [6] (TOCCATA)	2012	Prospective nonrandomized study	32	n/a	12	Circumferential PVI	TactiCath
Neuzil et al. [7] (EFFICAS I)	2013	Prospective nonrandomized study	46	n/a	3	Circumferential PVI plus remapping at 3 months	TactiCath
Casella et al. [8]	2014	Randomized controlled trial	20	35	12	Circumferential PVI	TactiCath or Contact Therapy Cool Path
Andrade et al. [9]	2014	Prospective nonrandomized study	25	50	13.3	Circumferential PVI	ThermoCool SmartTouch
Kimura et al. [10]	2014	Randomized controlled trial	19	19	6.7	Circumferential PVI	ThermoCool SmartTouch
Marijon et al. [11]	2014	Prospective nonrandomized study	30	30	12	Circumferential PVI	ThermoCool SmartTouch
Natale et al. [12] (SMART AF)	2014	Prospective nonrandomized study	161	n/a	6	Circumferential PVI plus possible linear ablations and CFAE. CTI line if patient with AFL	ThermoCool SmartTouch
Sciarra et al. [13]	2014	Prospective nonrandomized study	21	21	2.5	Circumferential PVI	ThermoCool SmartTouch
Wakili et al. [14]	2014	Prospective nonrandomized study	32	35	12	Circumferential PVI	TactiCath
Wutzler et al. [15]	2014	Prospective nonrandomized study	31	112	12	Circumferential PVI	TactiCath
Jarman et al. [16]	2015	Retrospective case–control study	200	400	11.4	PVI^a^	ThermoCool SmartTouch
Ullah et al. [1]	2014	Prospective nonrandomized study	50	50	12	PVI or WACA plus CTI plus mitral isthmus plus roof line^b^	ThermoCool SmartTouch
Kautzner et al. [17] (EFFICAS II)	2015	Prospective nonrandomized study	24	26 patients from EFFICAS I	3	Circumferential PVI plus remapping at 3 months	TactiCath
Sigmund et al. [18]	2015	Prospective case-matched control trial	99	99	12	Circumferential PVI plus linear ablation plus CFAE^c^	ThermoCool SmartTouch
Reddy et al. [19] (TOCCA-STAR)	2015	Randomized controlled trial	146	134	12	Circumferential PVI plus possible linear ablations and CFAE. CTI line if patient with AFL	TactiCath

^a^For paroxysmal atrial fibrillation (AF), additional linear ablation was performed only exceptionally; nonparoxysmal AF, use of additional lesions varied by operator, including linear lesions at the roof, mitral isthmus, posterior wall and CTI, targeting of complex fractionated electrograms, and ablation at the endocardial and epicardial aspects of the coronary sinus

^b^CTI line added in patients with AFL hx; if remained in AF, linear lesions added at mitral isthmus and roof, both point-to-point and drag

^c^PVI only, PVI with lines, PVI with lines and CFAE, and PVI with CFAE

*AFL* atrial flutter, *hx* history *n/a* not applicable, *PVI* pulmonary vein isolation, *SVT* supraventricular tachycardia, *CTI* cavotricuspid isthmus isolation, *CFAE* Complex Fractionated Atrial Electrograms, *WACA* wide antral circumferential ablation

Despite compelling evidence in atrial fibrillation ablation, no randomized controlled clinical trial has yet assessed the feasibility of CF sensing in the ablation of (paroxysmal) supraventricular tachycardia ((P)SVT). The relatively high success rate of SVT ablations using the conventional (non-CF-based) methodology might have contributed to this virtual ignorance of CF sensing for these procedures. The acute success rates of catheter ablation of atrioventricular nodal re-entry tachycardia (AVNRT) and of accessory pathway ablations (i.e. Wolff–Parkinson–White syndrome–atrioventricular re-entry tachycardia; WPW-AVRT) with the conventional (non-CF-based) approach has been reported to be 96–97% and 93% respectively, whereas the recurrence rate of AVNRT ablation is approximately 5% and that of WPW-AVRT ablation is around 8% [20–25]. Furthermore, in contrast to atrial fibrillation ablation, the ablation target in (P)SVT usually comprises a much smaller area of myocardial tissue (slow pathway, accessory pathways, cavotricuspid isthmus or an atrial focus). Another more practical reason for the virtual ignorance of CF sensing in (P)SVT might be the nonavailability of CF-sensing technology in conventional nonirrigated ablation catheters.

The aim of this randomized controlled trial (RCT) is to demonstrate the superiority of the CF-sensing-based approach compared to the conventional (non-CF-sensing) approach in the catheter ablation of PSVTs by assessing the improvement in the number of RF applications, and to prove the safety of the open-irrigated, CF-sensing ablation catheters.

## Methods/design

### Study design

**Fig. 1:**
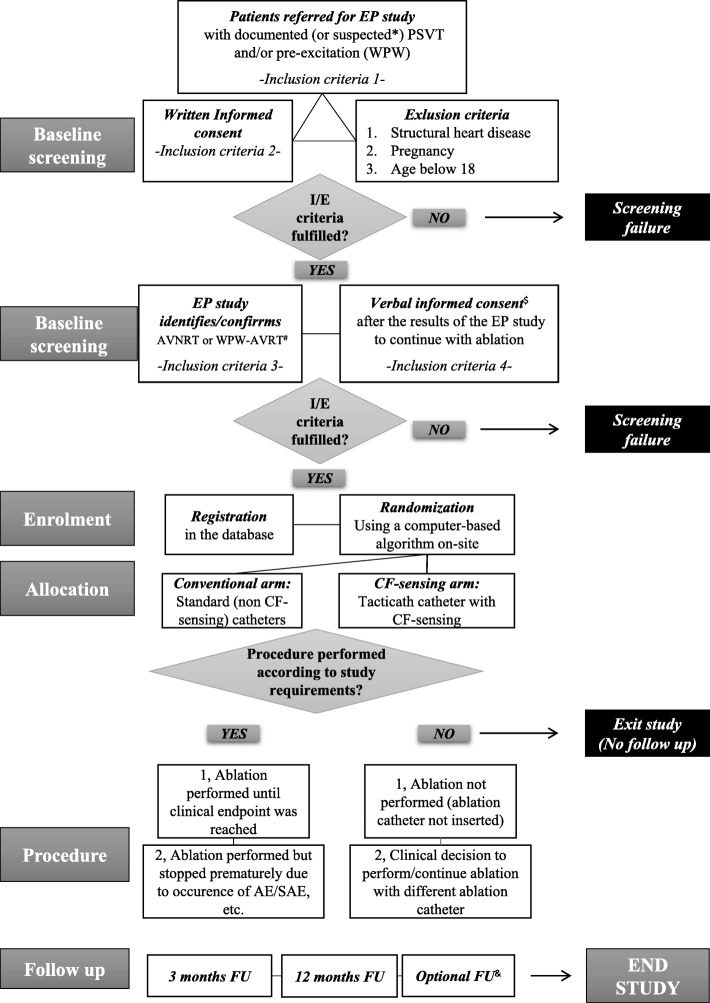
Contact-Force-Sensing-Based Radiofrequency Catheter Ablation in Paroxysmal Supraventricular Tachycardias (COBRA-PATH) study flow chart. *Symptoms highly suggestive of PSVT: sudden onset of termination of rapid (and regular) palpitations. The palpitations can usually be terminated with the Valsalva maneuver or carotid massage. The patients have no evidence for an underlying structural heart disease. ^#^For a detailed explanation, see “Study population”. ^$^For a detailed explanation, see “Preprocedural preparation and electrophysiology (EP) study”. ^&^This follow-up can be scheduled any time during the 12-month follow-up period, in case the patient experiences recurrent symptoms (symptom-based follow-up). AE adverse event, AVNRT atrioventricular nodal re-entry tachycardia, CF contact force, FU follow-up, I/E inclusion/exclusion, PVST paroxysmal supraventricular tachycardia, SAE serious adverse event, WPW-AVRT Wolff–Parkinson–White syndrome–atrioventricular re-entrant tachycardia

The COBRA-PATH study is a prospective, single-center, randomized controlled trial. Patients will be randomized to ablation using a standard (noncontact force-sensing) catheter or ablation using a contact force-sensing catheter (Fig. 1). The follow-up period is 12 months from the index procedure. All operators in the electrophysiology center of Erasmus MC have significant experience in (P)SVT ablation (more than 200 PSVT ablations). The protocol follows the Standard Protocol Items: Recommendations for Interventional Trials (SPIRIT) 2013 Statement. The SPIRIT 2013 Checklist is provided in Additional file 1. Enrolment, intervention and assessment according to the time schedule of the study are presented in the SPIRIT figure in Additional file 2.

### Study population

All patients without structural heart disease being referred to participating centers for standard electrophysiological study either because of pre-excitation detected on 12-lead surface ECG or because of palpitations associated with documented narrow-complex tachycardia (or clinical signs highly suggestive of the palpitation being associated with PSVT: sudden onset and termination of rapid and regular palpitations—the palpitations can usually be terminated with Valsalva maneuver or carotid sinus massage) will be eligible. In addition, in order to be eligible for participating in the trial, an electrophysiology study must identify AVNRT or WPW-AVRT (see clarification in the following) as the underlying arrhythmia mechanism [26–29]. In order to be included in the study, a subject must meet all of the following criteria:

- Referral for electrophysiology study because of pre-excitation on 12-lead surface ECG and/or documented (or suspected) symptomatic PSVT: palpitations associated with narrow-complex tachycardia (≥ 1 episode) documented by a 12-lead electrogram, Holter monitoring, trans-telephonic event recorder, telemetry strip or implanted device (implantable loop recorder, pacemaker) within 12 months prior to referral to EPS; or frequent symptoms of palpitation (within 12 months prior to referral) associated with clinical signs highly suggestive of PSVT—sudden onset and termination of rapid (and regular) palpitations usually accompanied with neck pulsation and/or dizziness, termination with Valsalva maneuver or carotid sinus massage, and no evidence for an underlying structural heart disease
- Identification of AVNRT or WPW-AVRT (with manifest or concealed accessory pathway) during standard EP study
- Verbal consent to continue with ablation therapy after the diagnostic steps of the EPS identified the aforementioned arrhythmia mechanisms, and after the patient received sufficient information about the benefits and potential risks of the ablative treatment of the individual arrhythmia substrate
- Informed written consent to being included in the study

A potential subject who meets any of the following criteria will be excluded from participating in this study:

- Evidence of structural heart disease and/or myocardial ischemia
- Pregnancy (and lack of exclusion of potential pregnancy)
- Age below 18 years

In this study protocol, we use the term WPW-AVRT to describe the following disease entities:

1. Patients with evidence of a manifest accessory pathway (i.e. pre-excitation on surface ECG and/or findings supporting anterograde conduction over an accessory pathway during standard EP study) who also present with documented narrow (or wide)-complex tachycardia, which proves to be orthodromic (or antidromic) AVRT by the EP study. (Note: antidromic AVRT is a quite rare entity.)
2. Patients presenting with documented orthodromic AVRT using a concealed bypass tract (accessory pathway conducting only in the retrograde direction).
3. Patients who possess a manifest accessory pathway (pre-excitation on ECG and/or evidence for anterograde conduction during EP study; similar to group 1) but have never experienced palpitations and have no prior documentation of tachycardia. In these patients, RF ablation of the accessory pathway will be offered for those individuals who possess a manifest accessory pathway with an anterograde ERP less than 240 ms, and/or for whom EP study identifies a reproducibly inducible AVRT. These patients can be enrolled in our study, even if no tachycardia had been experienced or documented in the past.

### Ethics

The study protocol was approved in September 2017 by the Medical Research Ethics Committee (2017-394) of Erasmus Medical Center in Rotterdam, the Netherlands. Patient inclusion began in June 2018 and is still ongoing. Written informed consent has and will be obtained from each participant.

### Primary and secondary outcome parameters

The primary outcome of this study is the number of radiofrequency ablations during the ablation procedures of AVNRT and WPW-AVRT. The secondary outcome parameters are overall duration of radiofrequency applications, acute procedural success/failure, fluoroscopy time, total procedural duration, (serious) adverse events and long-term procedural success. The following additional characteristics and parameters are collected and investigated during the study: baseline demographic characteristics of patients, previous antiarrhythmic drug treatment, history of cardiac arrhythmias (first onset of PSVT, and other possible associated arrhythmias, e.g. atrial fibrillation, atrial flutter, premature ventricular contraction, (non)-sustained VT) and prior ablation history.

### Study groups and randomization process

Consecutive patients on the waiting/referral list for PSVT ablations (referred to our tertiary center by other hospitals) will be screened for eligibility by dedicated study coordinators. When they meet the inclusion criteria, the patients will be enrolled in the study (as depicted in the flowchart for the study; see Fig. 1). After enrolment, patients will be allocated a unique study subject number in chronologically ascending order. Subsequently, they will be randomized to two treatment arms: the “CF-sensing” arm, ablation with a CF-sensing ablation catheter; and the “conventional” arm, standard ablation approach using a conventional (noncontact force-sensing) ablation catheter. Randomization will be performed by a computer-generated program (ALEA) based on a block randomization protocol using a block size factor of four. Randomization into the two treatment arms will be carried out with a 1:1 randomization ratio (50% probability for both treatment arms). The process of randomization will follow the general concepts of allocation concealment by incorporating the following prerequisites into the conduct of randomization: the EP specialists performing the ablation procedures do not have knowledge on the method of randomization utilized in the study; the EP specialists performing the ablation procedures do not keep a record of the results of randomization during previous ablation procedures; withdrawal from enrolment and randomization after the initial screening process and the subsequent EP study (that was positive for AVNRT or WPW-AVRT) must be explained thoroughly in the official clinical report by the treating specialist; the person in charge of the randomization process will not be the treating physician and neither will he/she be involved in the patient selection process; and the person in charge of the randomization will be contacted (over the telephone) by the treating specialist after the diagnostic part of the procedure has been completed. Stratification for study specific factors will not be conducted. The study will be performed in an open-label fashion: neither the patient nor the electrophysiologist (performing the procedure) will be blinded for the specific type of procedure.

### Electrophysiology procedure and catheter ablation

#### Preprocedural preparation and electrophysiology (EP) study

The procedures are performed under local anesthesia. In the case of a patient on anticoagulation therapy, this is decreased/discontinued before the procedure according to local guidelines. Antiarrhythmic medication is stopped 5 days before the procedure. The diagnostic EP study is implemented according to standard protocols for diagnosing SVTs: vascular access is created with femoral venous puncture and multipolar diagnostic catheters are placed in standard locations within the cardiac chambers: the coronary sinus, the right ventricle and the region of the bundle of His. Intravenous heparin is used during the procedure for anticoagulation (guided by the activated clotting time when necessary). Standard pacing maneuvers (atrial/ventricular extrastimulus testing, atrial/ventricular incremental pacing and specific pacing maneuvers to assess accessory pathway conduction and to induce and analyze SVTs) are performed to confirm the diagnosis of AVNRT or WPW-AVRT. After confirming the diagnosis of AVNRT/WPW-AVRT, the operator informs the patients about the specific diagnosis and briefly describes the expected success rates and safety concerns regarding the ablation of the particular subtype of arrhythmia substrate. Next, in order to continue with ablation, verbal consent is obtained from the patient (see protocol flowchart in Fig. 1).

#### Mapping and ablation of the underlying arrhythmia substrate

In the case of a left-sided ablation, the choice of access to the left atrium/ventricle (retrograde via the femoral artery vs. a transseptal approach) is left to the discretion of the operator. Transseptal punctures are guided by intracardiac echocardiography (ICE).

Depending on the results of the randomization, the subsequent steps of mapping/ablation are performed using either a conventional (nonirrigated, non-CF-sensing) manual RF ablation catheter (conventional arm) or the TactiCath Quartz open-irrigated contact force-sensing catheter (CF-sensing arm). Stable catheter–tissue contact (minimum of 10 s in duration) with a minimum CF exceeding 5–10 g (and an optimal CF within the range of 15–20 g) is targeted in the CF-sensing group. The TactiCath catheter is used in a virtual “nonirrigated mode” (saline flow set to ~ 1 ml/min) to achieve a similar degree of temperature control as in the case of conventional nonirrigated catheters. In both groups, the power (usually between 15 and 55 W) and the duration (usually between 60 and 90 s) settings of individual RF applications will follow local laboratory standards and will be left at the operator’s discretion. The ablation procedures are continued until the clinical tachycardia is no longer inducible and/or the conduction through an accessory pathway is terminated, provided that these clinical endpoints can be achieved with acceptable clinical risk–benefit ratios (e.g. no signs of imminent complete AV-block, no signs of pericardial effusion, etc.). Thereafter, the inducibility of the clinical tachycardia and/or conduction over an accessory pathway is reassessed throughout a waiting time of 30 min from the last RF application. No additional applications are implemented until further programmed stimulation shows evidence for recurrence of inducibility and/or re-conduction over an accessory pathway.

#### Exclusion of enrolled patients from further follow-up

Patients who were originally enrolled into the study (registered in the database and randomized to one of the two treatment arms) are excluded from further follow-up in the case of the following scenarios:

- The ablation catheter is not introduced into the patient due to prior occurrence of a serious AE (note: not during the ablation process itself), to the withdrawal of consent or to any type of technical difficulty with the setup.
- The ablation catheter is not introduced into the patient or ablation is discontinued before the achievement of acute success due to a clinical decision to utilize a different type of catheter that is not compatible with the study design (e.g. magnetic-navigation guided catheters, cryoablation catheters, etc.). Such clinical scenarios include, for example, parahisian accessory pathways (switching to cryoablation to prevent AV node injury), ablation of epicardial accessory pathways from a coronary sinus branch (magnetic navigation guided catheter for better access) or any other situation where the specific anatomy of the underlying arrhythmia-substrate involves an increased potential risk thereby necessitating the utilization of special approaches.

However, those individuals for whom ablation was implemented with a “study-compatible” ablation catheter yet the applications were prematurely discontinued due to the occurrence of (serious) AEs will indeed be taken into consideration for further analyses (i.e. these patients will not be excluded from further follow-up) in order to be able to assess the safety aspects of CF sensing vs*.* non-CF sensing during catheter ablation.

### Clinical follow-up procedure

Patients are discharged home within 2 days after the index procedure. Antiarrhythmic medication is discontinued after the ablation procedure. Follow-up of study patients is implemented as outlined in Table 2. All patients undergo physical examination, 12-lead ECG and echocardiography before and the next day after the procedure (note: only bedside echocardiography is performed after the procedure, in order to control for pericardial effusion). After discharge, patients have an outpatient visit at 3 months and at 12 months after the index procedure. During these outpatient visits, patients will be assessed for recurrence of palpitations (medical history), in addition to a physical examination and a 12-lead ECG. Additional outpatient visits during the 12-month follow-up period are scheduled only in the case of recurrence of symptoms (symptom-based follow-up appointments). In these cases, a detailed medical history and a 12-lead ECG will be taken, and the following additional examinations can also be performed (if necessary) in order to document the clinical arrhythmia: Holter monitoring (up to 3 weeks) and/or trans-telephonic event recorder (CardioMemo). In the case of recurrence, a redo electrophysiology study can be scheduled. An event qualifies as recurrence of clinical arrhythmia if the patient experiences identical complaints to those before the ablation procedure, and the same type of narrow-complex tachycardia is recorded with any of the following: 12-lead ECG, Holter monitor or trans-telephonic event recorder (CardioMemo). In addition, a recurrence is also identified in case the same type of clinical arrhythmia can be induced during a potential redo electrophysiology study, that was indicated due to recurrent palpitations. The occurrence of (serious) adverse events will be assessed and followed-up during the procedure, before discharge, and at 3 and 12 months post discharge (additional follow-up appointments can also be scheduled if necessary). Echocardiography is done in case follow-up of pericardial effusion/tamponade is necessary, chest X-ray is done to follow-up possible phrenic nerve damage and vascular ultrasound is done if vascular access complications require it.

**Table 2:** Follow-up design of the COBRA-PATH study

Type of clinical examination	Before the index procedure	1 day after the index procedure	3 months after the index procedure (outpatient visit)	12 months after the index procedure (outpatient visit)
Physical examination/history^a^	+	+	+	+
12-lead surface electrocardiogram^a^	+	+	+	+
Echocardiography	+	+ (bedside echo)	**–**	–
24-h (or longer-term) Holter monitoring^a^	If available	–	In case necessary to detect possible recurrence^b^	In case necessary to detect possible recurrence^b^
Trans-telephonic event recorder (CardioMemo)	If available	–	In case necessary to detect possible recurrence^b^	In case necessary to detect possible recurrence^b^

*COBRA-PATH* Contact-Force-Sensing-Based Radiofrequency Catheter Ablation in Paroxysmal Supraventricular Tachycardias

^a^These examinations can also be scheduled any time during the 12-month follow-up period in the case that the patient experiences recurrence of symptoms (symptom-based follow-up appointment)

^b^Decision to perform these examinations will be made jointly by the treating cardiologist and the study investigators

### Adverse events and safety monitoring

Adverse events are registered and reported according to regulations of the medical ethics committee. The occurrence of the following complications are considered a major (serious) adverse event: death, acute myocardial infarction/coronary artery damage, major bleeding, abdominal bleeding, cardiac tamponade or pericardial effusion requiring intervention (and/or prolonging the duration of hospitalization), ischemic cerebral event and other procedure-related embolic events, high-degree AV-block requiring PM implantation, atrial esophageal fistula, phrenic nerve palsy and vascular access complications (requiring intervention and/or prolonging the duration of hospitalization, e.g. arteriovenous fistula, or aneurysm). Minor adverse events are as follows: mild pericardial effusion, minor bleeding, and minor vascular access complications that do not require interventions and do not prolong the duration of hospitalization; in addition, nonsustained high-degree AV-block or sustained first-degree AV-block not requiring pacemaker implantation.

Since the conduct of this trial is considered low risk for participating subjects (due to the fact that CF-sensing catheters have already been proven by multiple clinical studies to be safe in the ablation of other types of cardiac arrhythmias), the ethical committee has agreed to omit the establishment of a safety committee.

### Statistical analysis

The number of radiofrequency applications during the ablation procedures of AVNRT and WPW-AVRT (primary study endpoints), as well as other continuous procedural parameters from the group of secondary study endpoints (such as total procedural duration, fluoroscopy time and overall duration of RF applications), will be expressed as mean ± SD or median (interquartile range) depending on their distributions, and statistical analysis of these variables will be conducted using either the two-sample *t* test or the Mann–Whitney test, again depending on the underlying distribution of the data.

The recurrence rate of PSVT at 1 year (as an indicator of long-term success rate of the index procedure), as well as the acute success rate and the major/minor complication rates of the index procedures (secondary study endpoints), will be presented as absolute and relative frequencies. The associations between treatment and these secondary endpoints will be analyzed with the chi-squared test or with Fischer’s exact test, as appropriate. Moreover, logistic regression will be performed to examine these associations and will, if needed, be adjusted for unbalanced baseline characteristics. Freedom from PSVT recurrence will be determined with Kaplan–Meier analysis, and differences in recurrence-free survival rates will be evaluated with the log-rank test. A *p* value of 0.05 will be considered significant for all statistical determinations, and all reported *p*-values will be based on two-sided tests.

### Sample size calculation

The sample size calculations were based on the following:

- A retrospective analysis of AVNRT/WPW-AVRT ablations performed in our institute within the last 6 months revealed that the mean number of radiofrequency applications required for successful ablations was 4.81 ± 3.21, which is in line with previous results found in the literature [30].
- According to previous studies, the ablation efficiency for patients with atrial fibrillation has been increased with the use of CF-sensing technology by approximately 15–20% [1, 4–19], as revealed by the reduced number and duration of RF applications in atrial fibrillation ablation. We assume that in terms of ablation efficiency, the significance of CF sensing will be even more pronounced in the treatment (catheter ablation) of such simple arrhythmia substrates as that of AVNRT and WPW-AVRT, therefore (regarding our sample calculations) a 25% reduction in RF application number was considered realistic to represent a clinically significant change.

Evaluation of the distribution of RF applications in our database of AVNRT/WPW-AVRT ablations (mentioned under the first point) revealed a positive skewness, and therefore logarithm transformation (natural log) was performed in order to approximate a normal distribution of the variables. The mean and standard deviation of the natural logs of application numbers were computed and amounted to 1.39 ± 0.59; these values were subsequently used in our sample size calculations.

For the sample size calculations, we used two different calculator tools: an online sample-size calculator tool (http://powerandsamplesize.com/Calculators/Compare-2-Means/2-Sample-Equality) and G*power (http://www.gpower.hhu.de/en.html). In both methods, we applied the appropriate test comparing the difference of two independent means of two groups (calculation formulas are presented on the calculator’s website and in G*power). The mean application number of the group with the study catheter was assumed to be reduced by 25% (as already mentioned) compared to the control group (thus amounting to 1.39 × 0.75 = 1.043) and the standard deviation of this group was assumed to be identical to the control group. The power was set to 80% and the type 1 error (α) to 5% (two-tailed). Using these parameters, both methods/calculators determined the same sample size of patients (2 × 46 = 92), which would be required to show a statistical significance in the case of a clinically significant reduction (25%, as mentioned earlier) of RF application number.

Allowing a dropout rate of 15%, the total sample size would become around 106. In addition, the acute success rate of AVNRT/WPW-AVRT ablations has been reported to be around 95% [26, 29]. Taking this into consideration, the total number of patients required for the purposes of this study would be 112.

## Discussion

In the past decades, radiofrequency catheter ablation has become firmly established as a first-line therapy of paroxysmal SVT, and according to European and US guidelines it has a Class I indication in symptomatic cases [26, 29]. The reported short-term and long-term success rates of the conventional approach of both AVNRT and WPW-AVRT ablations are reasonably high and they are also considered a safe procedure in terms of major/minor complication rates [20, 24, 30, 31]. The acute success rate of catheter ablation of AVNRT and WPW-AVRT with the conventional (non-CF-based) approach has been reported to be 96–97% and 93% respectively, whereas the recurrence rate of AVNRT ablation is approximately 5% and that of WPW-AVRT ablation is around 8% [20–25].

However, inadequate contact between the catheter tip and the target tissue during ablation might have an important role in cases where the arrhythmia substrate is difficult to ablate. This is especially true for such locations as the right free wall, where catheter-stability issues can significantly hinder accessory pathway ablation [30]. We expect that adding contact force-sensing technology to the conventional ablation approach can further improve acute and long-term success rates of AVNRT/WPW-AVRT ablations, and might also further improve the safety profile of these procedures. In terms of basic procedural parameters (RF application number/time, total procedural time, fluoroscopy time), we definitely expect an improvement compared with the conventional technology.

Our expectations are based on the results of several clinical trials which demonstrated that CF-sensing improves clinical outcomes in patients with atrial fibrillation in comparison with conventional catheter-based therapy [1, 4–19]. Table 1 presents an overview of these clinical studies. These investigations have demonstrated that CF-sensing improves the recurrence rate of atrial fibrillation in comparison to conventional catheter-based therapy, as well as significantly reducing the total procedure and ablation times. Moreover, CF-sensing allows operators to spend less time assessing signal or impedance drop during the ablation process. Exposure to radiation has also been reported to diminish with CF-sensing.

A recent meta-analysis of the controlled trials on CF-based ablation vs. conventional approach in patients with atrial fibrillation (presented in Table 1) has found enhanced safety of using CF-based ablation technology with acceptable rates of minor and major complications and reduced rates of cardiac perforation (although this did not reach statistical significance) [3]. This is probably attributable to two factors: the continuous monitoring of the catheter while manipulating the cardiac chambers, with real-time feedback of catheter tip–tissue contact; and avoiding ablation when the CF is suboptimal (which would reduce the need for excessive ablations and subsequent related complications). We strongly believe that through these two factors, CF sensing would have a similar beneficial effect on the complication rate of AVNRT/accessory pathway ablation. Therefore (based on the aforementioned studies), as far as potential risks associated with CF sensing are concerned, we believe that there is no additional risks of complications expected with the use of CF sensing during AVNRT/accessory pathway ablation. Instead, using CF sensing should rather reduce the complication rate and enhance the safety profile of these ablations, as well.

Despite compelling evidence in atrial fibrillation ablation, no randomized controlled clinical trial has yet assessed the feasibility of CF sensing in the ablation of (P)SVTs. Although the TOCCATA study enrolled patients with right-sided SVTs as well, it was conducted in a nonrandomized, uncontrolled fashion, comparing the results of CF-sensing-based catheter ablations (performed in this study with the TactiCath catheter) with the results of conventional (non-CF-sensing-based) ablation techniques reported in the literature (for right-sided SVT) prior to the study. In addition, the focus of this trial was the safety profile of the CF-sensing catheter, and parameters such as the recurrence rate, total procedure/ablation time and fluoroscopy time have not been evaluated for right-sided SVTs [5, 6].

Since catheters equipped with CF-sensing technology already have an established role in atrial fibrillation ablation, and are also demonstrated to represent a safe methodology, we raise no potential concerns regarding the usage of the same types of catheters in the population of patients with PSVT. On the contrary, we strongly believe that the aforementioned results of the CF-sensing technology in AF ablations are also highly suggestive for the potential of this technology to be beneficial for PSVT patients. Since clear evidence (based on randomized controlled clinical trials) on the superiority of this technology in PSVT patients is currently lacking, we believe that our study can give an answer to the question of whether CF sensing is beneficial to use in ablation procedures targeting less complex substrates, like the slow-pathway of the AV node and the accessory pathways in different anatomic locations.

### Limitations

Commercially available CF-sensing catheters utilize irrigated-tip technology as a standard feature (and currently, nonirrigated CF-sensing catheters are not being manufactured). However, the standard catheter type used for PSVT ablations is a nonirrigated catheter. In order to create a comparable ablation approach, we intend to use the CF-sensing catheter in a “virtual nonirrigated modus” (irrigation rate set at 2 ml/min), thereby achieving similar temperature-controlled ablation characteristics to those with the nonirrigated catheters. The small amount of residual irrigation could, however, lead to potential bias and inconsistencies as far as the homogeneity of the two ablation approaches are concerned, thereby adding another possible non-negligible factor to the equation, which can have a potential effect on outcome and efficacy. However, based on our experience (gained with several commercially available irrigated-tip catheters throughout a significant number of ablations in our EP center), the efficacy and safety profiles of an irrigated catheter utilized in a “nonirrigated modus” are comparable to the characteristics of nonirrigated ablation.

## Trial status

Recruitment of participants started on 25 August 2017 and is expected to be completed on 30 June 2020. This manuscript is based on protocol version 02 of 25 August 2017.

## Supplementary information

**Additional file 1.** SPIRIT 2013 Checklist.

**Additional file 2.** SPIRIT figure.

## Data Availability

The datasets used and/or analyzed during the current study are available from the corresponding author on reasonable request.

## Abbreviations

AE: Adverse event
AV-block: Atrioventricular block
AVNRT: Atrioventricular nodal re-entry tachycardia
CF: Contact force
EPS: Electrophysiology study
ERP: Effective refractory period
ICE: Intracardiac echocardiography
(P)SVT: (Paroxysmal) Supraventricular tachycardia
RF: Radiofrequency
VT: Ventricular tachycardia
WPW-AVRT: Wolff–Parkinson–White syndrome–atrioventricular re-entrant tachycardia
