# Supplementary material for: Contact-Force-Sensing-Based Radiofrequency Catheter Ablation in Paroxysmal Supraventricular Tachycardias (COBRA-PATH): a randomized controlled trial
Source: Trials. 2020 Apr 9;21:321. doi: 10.1186/s13063-020-4219-1 (PMC7147009; doi:10.1186/s13063-020-4219-1)
Supplement: Supplementary file 2 — Additional file 2. SPIRIT figure. [file 13063_2020_4219_MOESM2_ESM.doc]

***Additional file 2.:*** Spirit Figure

|  | **STUDY PERIOD** | | | | | | |
| --- | --- | --- | --- | --- | --- | --- | --- |
|  | **Enrolment** | **Allocation** | **Post-allocation** | | | | **Close-out** |
| **TIMEPOINT**** | ***-t_1_*** | **0** | ***Procedure*** | ***3 months FU*** | ***12 months FU*** | ***Optional FU*** | ***t_x_*** |
| **ENROLMENT:** |  |  |  |  |  |  |  |
| **Eligibility screen** | X |  |  |  |  |  |  |
| **Informed consent** | X |  |  |  |  |  |  |
| **Allocation** |  | X |  |  |  |  |  |
| **INTERVENTIONS:** |  |  |  |  |  |  |  |
| ***[Contact-force sensing ablation]*** |  |  | X |  |  |  |  |
| ***[Conventional ablation]*** |  |  | X |  |  |  |  |
| **ASSESSMENTS:** |  |  |  |  |  |  |  |
| ***List baseline variables*** | X |  |  |  |  |  |  |
| ***[Primary outcome parameters]*** |  |  |  |  |  |  |  |
| ***Number of RF applications*** |  |  | X |  |  |  |  |
| ***[Secondary outcome parameters]*** |  |  |  |  |  |  |  |
| ***Overall duration of RF applications*** |  |  | X |  |  |  |  |
| ***Long-term procedural success*** |  |  |  |  | x |  |  |
| ***Acute procedural success/failure*** |  |  | X |  |  |  |  |
| ***Fluoroscopy time*** |  |  | X |  |  |  |  |
| ***Total procedural duration*** |  |  | X |  |  |  |  |
| ***(serious) adverse events*** |  |  | x | x | x | x |  |
| ***Time to recurrence of AVNRT/WPW-AVRT*** |  |  | x | x | x | x |  |

*t_1_ : Procedure
t_2_ : After procedure, before discharge
t_3_ : Follow up 3 months
t_4_ : Follow up 12 months*
